# Correction to: Clustering spatial transcriptomics data

**DOI:** 10.1093/bioinformatics/btad574

**Published:** 2023-09-20

This is a correction to: Haotian Teng, Ye Yuan, Ziv Bar-Joseph, Clustering spatial transcriptomics data, *Bioinformatics*, Volume 38, Issue 4, February 2022, Pages 997–1004, https://doi.org/10.1093/bioinformatics/btab704

In the originally published version of this manuscript, the incorrect version of the Supplementary material was linked to the article. The supplementary material has now been made available as a PDF.

This error has been corrected online.

